# Evaluation of fixed dose four-factor prothrombin complex concentrate for warfarin reversal at a level 1 trauma center

**DOI:** 10.1186/cc14426

**Published:** 2015-03-16

**Authors:** H Drone, J Jancik, J Gorlin, M McCarthy

**Affiliations:** 1 Hennepin County Medical Center, Minneapolis, MN, USA

## Introduction

FDA-approved dosing of four-factor prothrombin complex concentrate (4F-PCC) in the USA is based on an INR and weight; however, there are data suggesting that a fixed dose of 4F-PCC may be sufficient for INR reversal and hemostasis [1,2]. The objective of this study was to assess the efficacy and safety of a fixed dose of 1,500 units of 4F-PCC. Historically, warfarin reversal included a combination of factor IX complex, vitamin K, and fresh frozen plasma (FFP). Using a fixed dose of 4F-PCC may also provide significant cost savings when compared with traditional dosing.

## Methods

This retrospective chart review compared 26 admitted adults who received a fixed dose of 1,500 units 4F-PCC with 26 patients who received a combination of factor IX complex and vitamin K, with or without FFP, for warfarin reversal from 1 January 2012 to 1 November 2014. Primary outcomes included reversal to an INR of <2 and reversal to an INR of <1.6. Secondary outcomes included ICU and hospital length of stay (LOS), change in INR, INR nadir, potential cost savings from 4F-PCC versus traditional dosing, and major adverse effects.

## Results

The INR was reduced to <2 in 100% of patients in the 4F-PCC group versus 84.6% of patients in the factor IX group (*P *< 0.05). The INR was reduced to <1.6 in 90.8% of patients in the 4F-PCC group versus 50% in the factor IX group (*P *< 0.05). Mean pre-reversal INRs were 3.5 and 4 and ranged from 1.1 to 10 and from 1.3 to 10 in the 4F-PCC and factor IX group respectively (*P *= 0.29). On average, a medication cost savings of US$802.63 dollars per patient was calculated from using a fixed 1,500 unit dose over traditional dosing of 4F-PCC. There was a trend toward a shorter mean ICU LOS in the PCC group when compared with the factor IX group (5.8 vs. 2.8 days) and shorter mean hospital LOS (10.7 vs. 5.7 days), although neither outcome was statistically significant. No difference in adverse event rates was observed.

## Conclusion

A fixed dose of 1,500 units of 4F-PCC was significantly more effective at lowering the INR to a threshold of less than either 2 or 1.6 when compared with a combination of factor IX complex and vitamin K with or without FFP. Further research is needed to investigate clinical outcomes and a possible reduction in ICU and hospital LOS.
